# Response of Human Thalamic Neurons to High-Frequency Stimulation

**DOI:** 10.1371/journal.pone.0096026

**Published:** 2014-05-07

**Authors:** Merrill J. Birdno, Wei Tang, Jonathan O. Dostrovsky, William D. Hutchison, Warren M. Grill

**Affiliations:** 1 Department of Biomedical Engineering, Duke University, Durham, North Carolina, United States of America; 2 Department of Electrical and Computer Engineering, Duke University, Durham, North Carolina, United States of America; 3 Department of Neurobiology, Duke University, Durham, North Carolina, United States of America; 4 Department of Surgery, Duke University, Durham, North Carolina, United States of America; 5 Department of Physiology, University of Toronto, Toronto, Ontario, Canada; 6 Toronto Western Research Institute, Toronto, Ontario, Canada; McGill University, Canada

## Abstract

Thalamic deep brain stimulation (DBS) is an effective treatment for tremor, but the mechanisms of action remain unclear. Previous studies of human thalamic neurons to noted transient rebound bursting activity followed by prolonged inhibition after cessation of high frequency extracellular stimulation, and the present study sought to identify the mechanisms underlying this response. Recordings from 13 thalamic neurons exhibiting low threshold spike (LTS) bursting to brief periods of extracellular stimulation were made during surgeries to implant DBS leads in 6 subjects with Parkinson's disease. The response immediately after cessation of stimulation included a short epoch of burst activity, followed by a prolonged period of silence before a return to LTS bursting. A computational model of a population of thalamocortical relay neurons and presynaptic axons terminating on the neurons was used to identify cellular mechanisms of the observed responses. The model included the actions of neuromodulators through inhibition of a non-pertussis toxin sensitive K^+^ current (I_KL_), activation of a pertussis toxin sensitive K^+^ current (I_KG_), and a shift in the activation curve of the hyperpolarization-activated cation current (I_h_). The model replicated well the measured responses, and the prolonged inhibition was associated most strongly with changes in I_KG_ while modulation of I_KL_ or I_h_ had minimal effects on post-stimulus inhibition suggesting that neuromodulators released in response to high frequency stimulation are responsible for mediating the post-stimulation bursting and subsequent long duration silence of thalamic neurons. The modeling also indicated that the axons of the model neurons responded robustly to suprathreshold stimulation despite the inhibitory effects on the soma. The findings suggest that during DBS the axons of thalamocortical neurons are activated while the cell bodies are inhibited thus blocking the transmission of pathological signals through the network and replacing them with high frequency regular firing.

## Introduction

High frequency deep brain stimulation (HFS) in the ventralis intermedius (Vim) nucleus of the thalamus is an established therapy for the treatment of tremor in movement disorders, including essential tremor (ET) and Parkinson's disease (PD). Although the clinical benefits of HFS are well documented, fundamental questions remain about the mechanisms of action. A hallmark of the effects of thalamic stimulation on tremor is the sensitivity to stimulation frequency (reviewed in [1]). Maximum tremor suppression is typically observed only when the frequency of stimulation is greater than 90 Hz, and lower frequencies are ineffective or may exacerbate symptoms [2], [3], [4]. Little is known about the responses of human thalamic neurons to stimulation with different parameters. To gain a better understanding of possible mechanisms that may underly neuronal responses to thalamic DBS, in the present study we recorded extracellularly the responses of human thalamic neurons in human subjects following brief periods of extracellular stimulation and subsequently used computer simulations in a biophysical model of thalamic stimulation to identify candidate cellular and ionic mechanisms that might explain the observed responses.

The post-stimulus responses of human thalamic neurons to brief periods of HFS included a short period of bursting followed by longer duration cessation of neuronal firing. The relatively long duration of these changes in neuronal firing after the cessation of stimulation suggested the influence of neuromodulators as a candidate mechanism for these effects, since GABA release is unlikely to explain such long lasting effects. For example, adenosine levels increased during intervals of HFS in thalamic slices and intrathalamic infusion of adenosine suppressed tremor in the harmaline mouse model of tremor [5]. Similarly, activation of the hyperpolarization-activated cation current (I_h_) by glutamate suppressed spindle oscillations in a thalamic slice [6]. Other potential neuromodulators include histamine, acetylcholine, serotonin, and noradrenaline. These neuromodulators can activate a pertussis toxin sensitive potassium current (I_KG_) [7], [8], inhibit a non-pertussis toxin sensitive leak potassium current (I_KL_) [9], [10], and/or shift the activation curve of I_h_ [11], [12].

Results of the computer simulations in the present study indeed indicate that neuromodulators likely play an important role in the post-stimulus responses of thalamic neurons to extracellular stimulation. The modulation of I_KG_ was necessary to produce the long pauses that occurred after stimulation, and modulation of I_KL_ was important for evoking the short epochs of bursts that preceded the long pauses. Despite the effects of neuromodulators on the *post-stimulus* behavior of thalamic neurons, the axons of the model thalamocortical relay neurons responded robustly to suprathreshold stimulation in the period *during* stimulation, and activity in these axons was driven by the stimulation pulses (regularized) during HFS. These results suggest that the effects of neuromodulators released in response to HFS are responsible for the post-stimulation bursting and subsequent long inhibition of firing of thalamic neurons. The influence of neuromodulators on the output of thalamic neurons *during* HFS is likely to be masked by direct activation of their axons, but may contribute to disruption of transmission of pathological activity (e.g., tremor-related oscillatory firing).

## Methods

### Ethics statement

The University Health Network Ethical Review Board at the University of Toronto approved all procedures, and participants gave written informed consent.

### Human thalamic recordings

Responses to extracellular stimulation were recorded intraoperatively from 17 anterior motor thalamic (Voa, Vop, Vim) and reticular nucleus neurons exhibiting low threshold spike (LTS) bursting in 6 awake human subjects with PD. We examined only cells that exhibited burst firing, because from previous observations we knew that cells exhibiting this type of activity were particularly sensitive to low intensity microstimulation, i.e., would reliably and consistently display a stereotypical pattern of protracted inhibition followed by return of bursting activity. In a previous study from our group we found that 81% of bursting cells and 24% of non-bursting cells were inhibited by thalamic HFS (Patra, MSc thesis, University of Toronto). The recordings were conducted during surgery to place deep brain stimulation (DBS) leads in the subthalamic nucleus (STN). Subjects were taken *off* of their dopaminergic medications for at least 12 hours prior to surgery.

A tungsten microelectrode was used for both extracellular stimulation and recording (10 Hz–5 kHz bandpass filtered, 1000–5000 gain; Guideline 3000 system, Axon Instruments, Union City, CA) in each subject (see [13] for further details). The microelectrode passed through the thalamus as it was driven toward the STN. While the microelectrode was in the anterior motor area of the thalamus, short-duration trains of extracellular stimulation were delivered through the microelectrode. Low-threshold spiking (LTS) thalamic neurons were targeted for recording [14]. The parameters of stimulation included variable train durations (0.5–2 s) and frequencies (0–200 Hz), as well as amplitudes (1–100 µA, but in most cases 1–5 µA). Recordings were processed using Spike 2 software (Cambridge Electronics Devices, Cambridge, UK), with further details provided in previous publications [15], [16], [17]. Because stimulation was delivered through the same electrode used for recording, it was not possible to record responses of the thalamic neurons during stimulation.

### Computational model

We implemented and validated a biophysically based computational model of a thalamic neuronal network to simulate the responses of anterior motor thalamocortical (TC) neurons of the thalamus to microstimulation. Each of 24 TC neurons was represented with a cable model that we previously developed and validated [18], [19]. The model TC neuron had a geometry obtained from a 3-D reconstruction of a filled cell [20] and reproduced a wide range of experimental electrophysiology. Presynaptic axons were modeled using a double cable representation of mammalian axons, which we previously developed and validated [21]. Subsequently, each element that was added to the model was based on biological data, and the integrated model was thoroughly validated against available data (File S1; 19). We used the model to calculate the combined effects of the intrinsic synaptic inputs and microstimulation on TC neuron activity. Thus, the responses of each TC neuron depended on a combination of the intrinsic synaptic inputs, changes in the intrinsic synaptic activity evoked by stimulation of presynaptic axons, and the direct effects of stimulation on the TC neuron.

**Figure 1:**
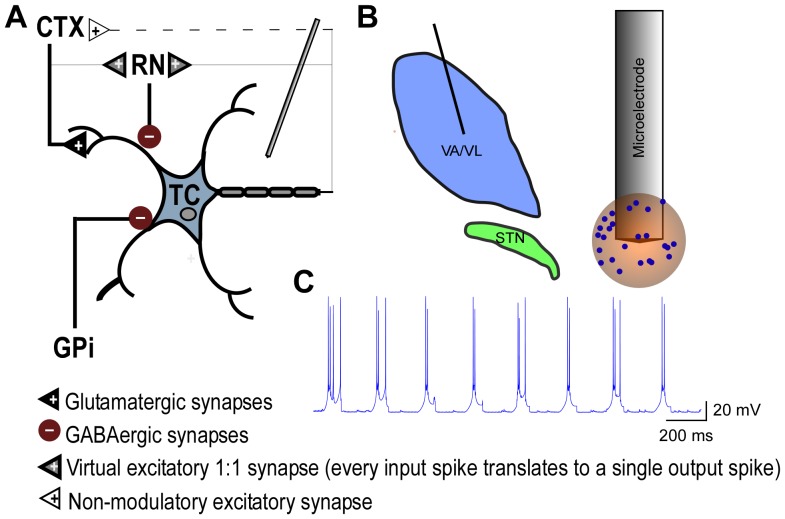
Computational model of thalamic stimulation. **a**. Schematic of three-dimensional cable model of a thalamocortical (TC) neuron and three terminating axons providing input to the TC neuron. Elements with bold lines were biophysically-modeled and subjected to the extracellular potentials generated by stimulation, while elements with light lines were modeled as virtual axon branches that mimicked activity in the biophysical axons with a time delay consistent with action potential propagation down an axon branch (modified from Birdno et al., 2012). **b**. Schematic of the thalamus and subthalamic nucleus, as well as an expanded view of the tip of the model microelectrode and sample 3-D locations of cell bodies surrounding the microelectrode. **c**. Intrinsic LTS bursting in model TC neuron in the absence of stimulation.

The fundamental unit of the model (**Fig. 1**) consisted of a TC neuron and three terminating axons carrying inputs from the globus pallidus internus (GPi), cortex (CTX), and reticular nucleus (RN) [22], [23], and this unit was replicated 24 times to simulate the response of a distributed population of neurons. Each input had intrinsic activity, and the inputs were distributed across the compartments of the TC neuron based on reconstruction of synaptic inputs to cat ventral thalamic neurons [18], [24]. The intrinsic activity of CTX inputs was a 20 Hz Poisson train of spikes [25], while the intrinsic activity of GPi inputs consisted of bursts with the following characteristics: spikes/burst  = 7; intraburst interspike interval  = 10 ms; interburst interval  = 170 ms [26]. The intrinsic activity of inhibitory RN inputs resulted from synaptic excitation of RN by CTX and synaptic excitation of RN via feedback from the TC output [27], [28]. The intrinsic inputs and dynamics of the model TC neuron resulted in LTS-like bursting in the absence of stimulation (**Fig. 1c**).

### Model geometry and extracellular stimulation

The center of the cell body of each model TC neuron and its input axons were positioned within a 600 µm diameter sphere centered on the microelectrode by generating uniformly distributed random coordinates (**Fig. 1b**). The extracellular voltages produced by microstimulation were calculated using a finite element model representation of the tungsten microelectrode in an isotropic homogeneous medium with conductivity σ = 0.2 S/m, and the conductivities of the metal electrode and insulating material were 1e7 and 1e–10 S/m, respectively [29]. The voltages in the modeled tissue volume were computed using COMSOL Multiphysics 3.4 (COMSOL, Burlington, MA). All simulations were performed with the microelectrode as the cathode, and the outside faces of a 20 cm ×20 cm ×20 cm cube were set to ground to approximate the counter electrode at a long distance from the stimulation site. The boundary condition on the surface of the microelectrode was set to constant voltage, while continuity of current density normal to the surface was imposed over the insulated portion. Voltages outside each compartment of each model TC neuron and each presynaptic axon were calculated using quadratic interpolation of the voltages at the grid points of the finite element mesh.

### Simulation methods

The model neurons were implemented in NEURON 6.1 [30], and the transmembrane potential in response to stimulation was obtained by backward Euler implicit integration with a time step of 0.01 ms. The population of neurons was simulated for a total of 15 s: 2 s before stimulation, 0.5–2 s during stimulation, and 11–12.5 s after stimulation. A range of stimulus amplitudes, frequencies, and durations corresponding to those used in the human subjects were tested in the model. All data presented from the model represent the responses of the distal end of the model thalamic axon(s).

### Analysis methods

The mean normalized autopower spectrum of each neuron was computed to quantify the bursting behavior exhibited by the human and model neurons. The autocorrelation of the spike times was calculated for each neuron, and then the power spectrum of the autocorrelation (autopower spectrum) was determined. The autopower for each neuron was normalized and then the mean and s.e.m. of the autopower spectra were computed across the populations of 17 human and 24 model neurons.

Results are reported as mean ± s.e.m., and statistical significance was defined at α = 0.05. Statistical differences were determined using analysis of variance (ANOVA) performed using the anova1 function in MATLAB (one-way ANOVA, Mathworks Inc., Natick, Massachusetts, USA), followed by Bonferroni multiple comparisons tests made using the multcompare function (multiple comparison test, Bonferroni correction) in MATLAB.

## Results

The predominant response of human thalamic neurons immediately after cessation of HFS included a short epoch of burst activity (median duration  = 1.25 s), followed by a prolonged period of silence (mean ± s.e.m.  = 4.02±0.42 s, for all trials with HFS frequency >90 Hz), before a return to LTS bursting.

### Pooled responses to short-duration, high-frequency stimulation

**Figure 2:**
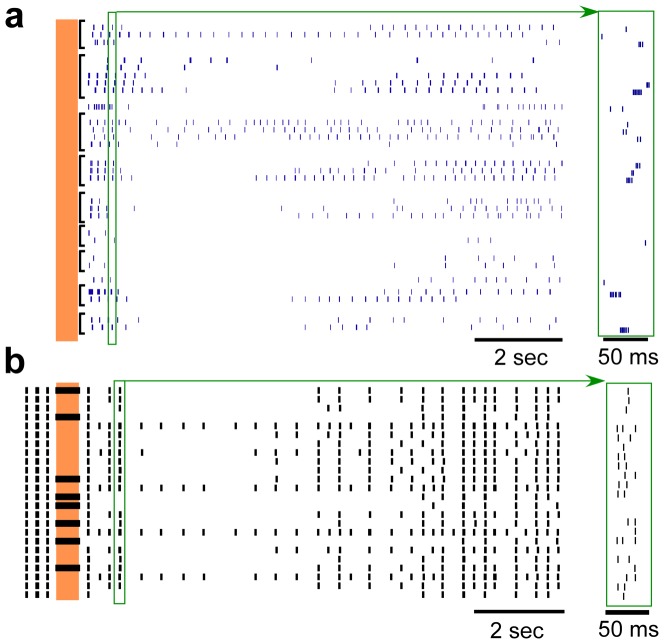
Post-stimulus responses of multiple neurons to brief epoch of high-frequency stimulation. (**a**) Rasters of repeated trials across 9 different human thalamic neurons to 0.5 seconds of stimulation at 200 Hz and with stimulus intensities between 1.5–5 µA. (**b**) Single trial rasters across the 24 model neurons in response to 0.5 seconds of stimulation at 200 Hz and with a stimulus intensity of 1.5 V. The vertical shaded region indicates the time when stimulation was on. Insets to the right are expanded in the time axis to illustrate sample individual action potentials within burst episodes. The characteristic post-stimulus bursting followed by prolonged inhibition is present in both human and model neuron recordings. Note that the spontaneous bursting activity prior to stimulation is not shown in (a).

The majority of human thalamic neuron recordings were in response to HFS with a duration of 0.5 s, frequency of 200 Hz, and amplitude >1 µA. Raster plots of repeated responses across 9 human neurons and single trials across 24 model thalamic neurons to stimulation at 200 Hz for 0.5 s are shown in **Figure 2**. The predominant post-stimulus response, including a brief period of bursting followed by a prolonged period of inhibition, was robust across these populations of human and model neurons. The mean durations of post-stimulus bursting in the human and model responses were 1.46±0.20 s and 1.08±0.17 s, respectively (mean ± s.e.m.), and the difference did not reach statistical significance (p = 0.16, unpaired t-test, T50  = 1.41). The mean durations of the prolonged periods of inhibition in the human and model responses were 5.0±0.51 s and 4.3±0.45 s, respectively (p = 0.30, unpaired t-test, T50  = 1.04).

### Burst oscillations

**Figure 3:**
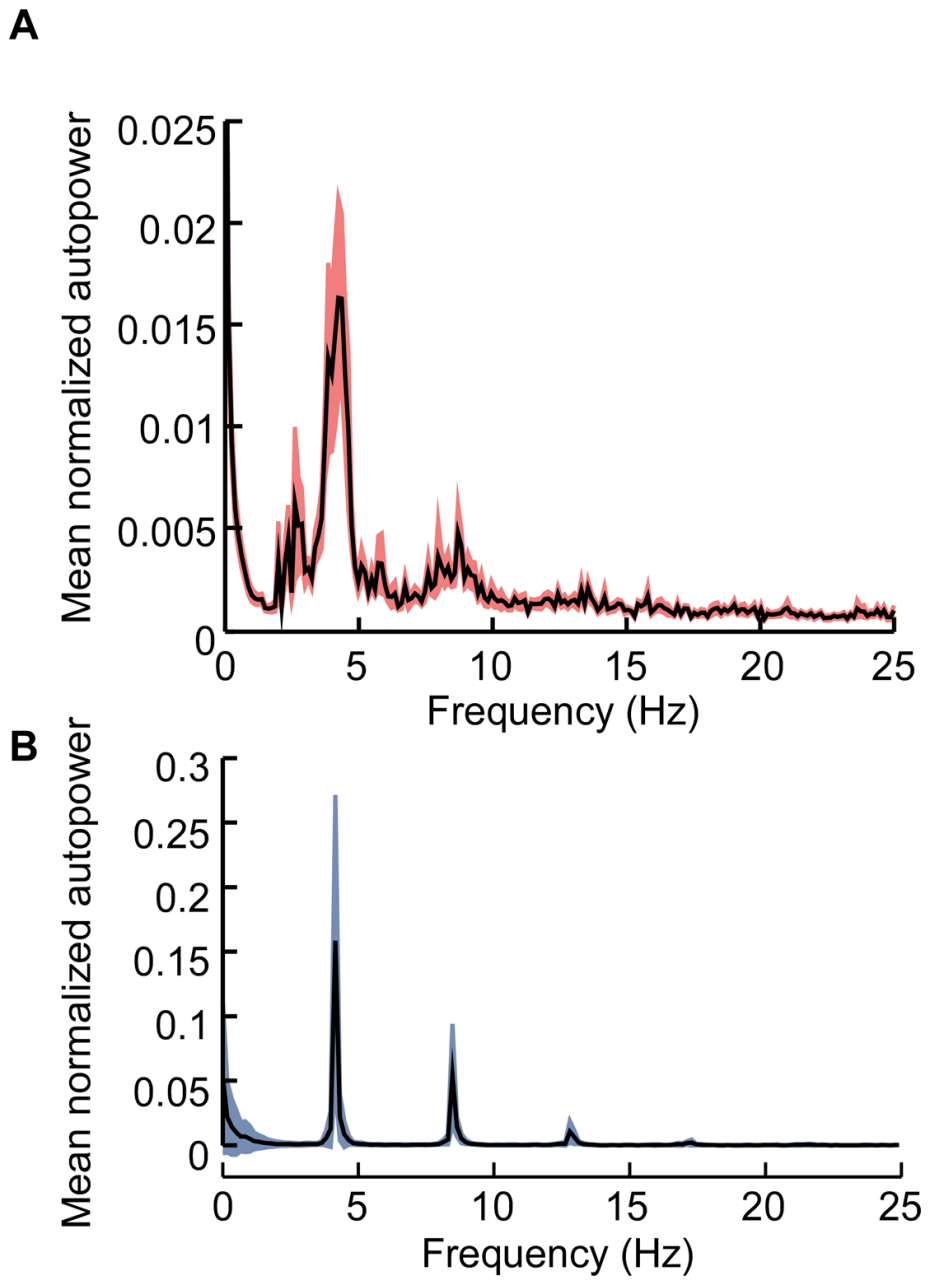
Burst oscillations in human and model thalamic neurons. **a**. Mean normalized autopower spectra of the human thalamic neurons. **b**. Mean normalized autopower spectra of the model thalamic neurons. Traces shown mean ± s.e.m.

The mean normalized autopower of the model neuron spike times paralleled closely the mean normalized autopower spectra of the human thalamic neuron activity (**Fig. 3**). Bursting in human neurons (spikes/burst  = 3.0±0.2, intraburst interspike intervals  = 5.1±1.1 ms) was at 4.23 Hz (peak in mean normalized autopower, n = 53, **Fig. 3**), and bursting in model neurons (spikes/burst  = 1.5±0.05, intraburst interspike intervals  = 12.2±0.1 ms) was at 4.15 Hz.

### Individual neuronal responses to varying stimulus parameters

**Figure 4:**
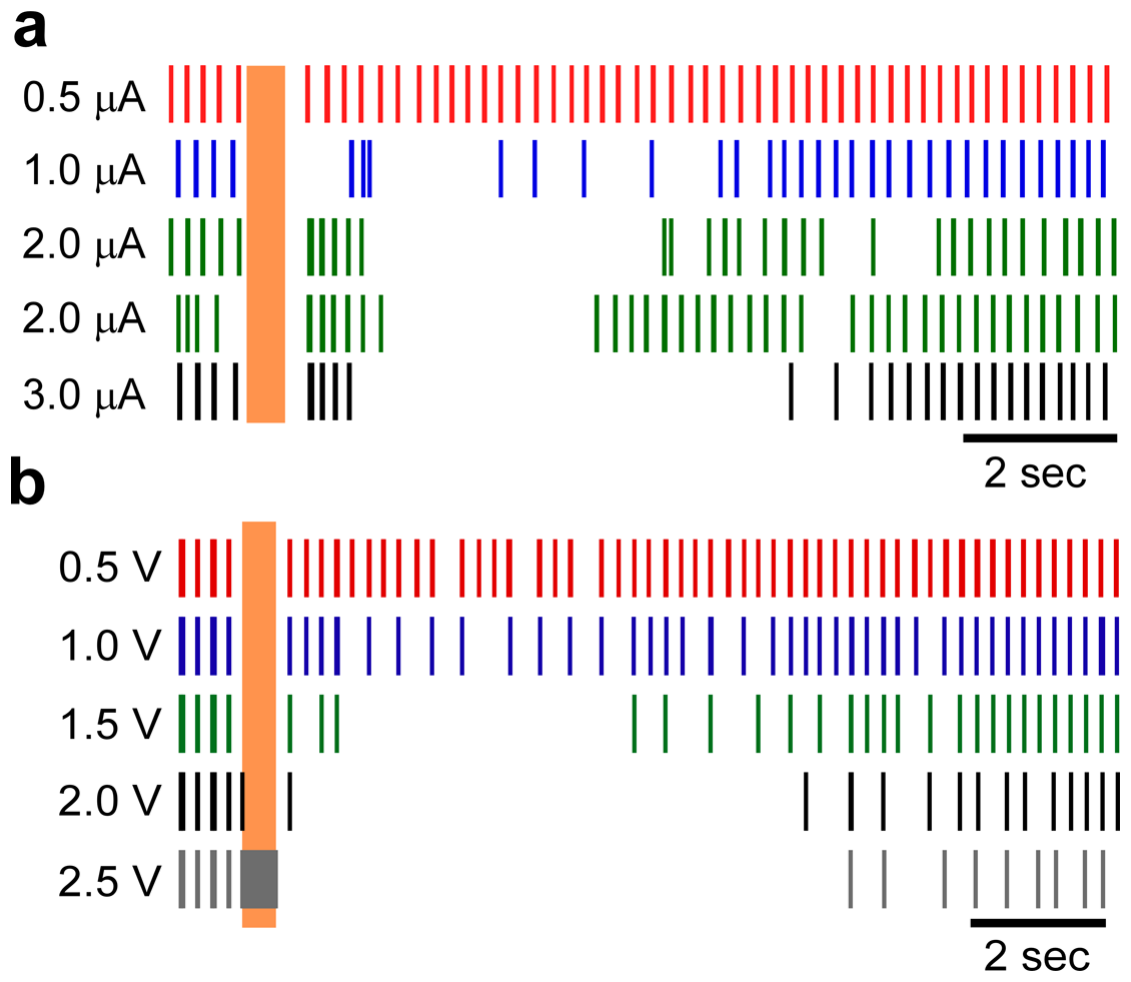
Individual neuron responses to changes in stimulus amplitude. Rasters of an individual human thalamic neuron (**a**) and an individual model neuron (**b**) to various amplitudes of stimulation at 200 Hz for 0.5 seconds. Replicate trials of 2.0 µA stimulation are shown to illustrate the trial-to-trial variability in the responses of the human neurons. The stimulus amplitude for each raster is indicated at the left. The vertical shaded region indicates the time when stimulation was on.

We measured changes in individual neuronal responses to changes in the amplitude of high frequency extracellular stimulation, while the frequency and duration of stimulation were held constant. The post-stimulus response of human thalamic neurons was muted with stimulation at low amplitudes, but became more robust as the amplitude of stimulation increased up to 5 µA (**Fig. 4a**). As the stimulus amplitude was increased, the duration of the post-stimulus inhibitory period likewise increased (linear least squares regression, human: *p*<0.03, R^2^ = 0.15; model: *p*<0.0001, R^2^ = 0.71). However, the duration of the initial rebound burst response did not vary significantly across stimulus amplitudes (*p* = 0.65, linear least squares regression, R^2^ = 0.006). Responses in the model neurons to increases in stimulus amplitude paralleled closely those observed in the human neurons, with one exception (**Fig. 4b**). The initial post-stimulus burst response was muted in the model neurons as the stimulus amplitude increased (*p*<0.0001, linear least squares regression, R^2^ = 0.73), whereas this was not observed in the human neurons.

**Figure 5:**
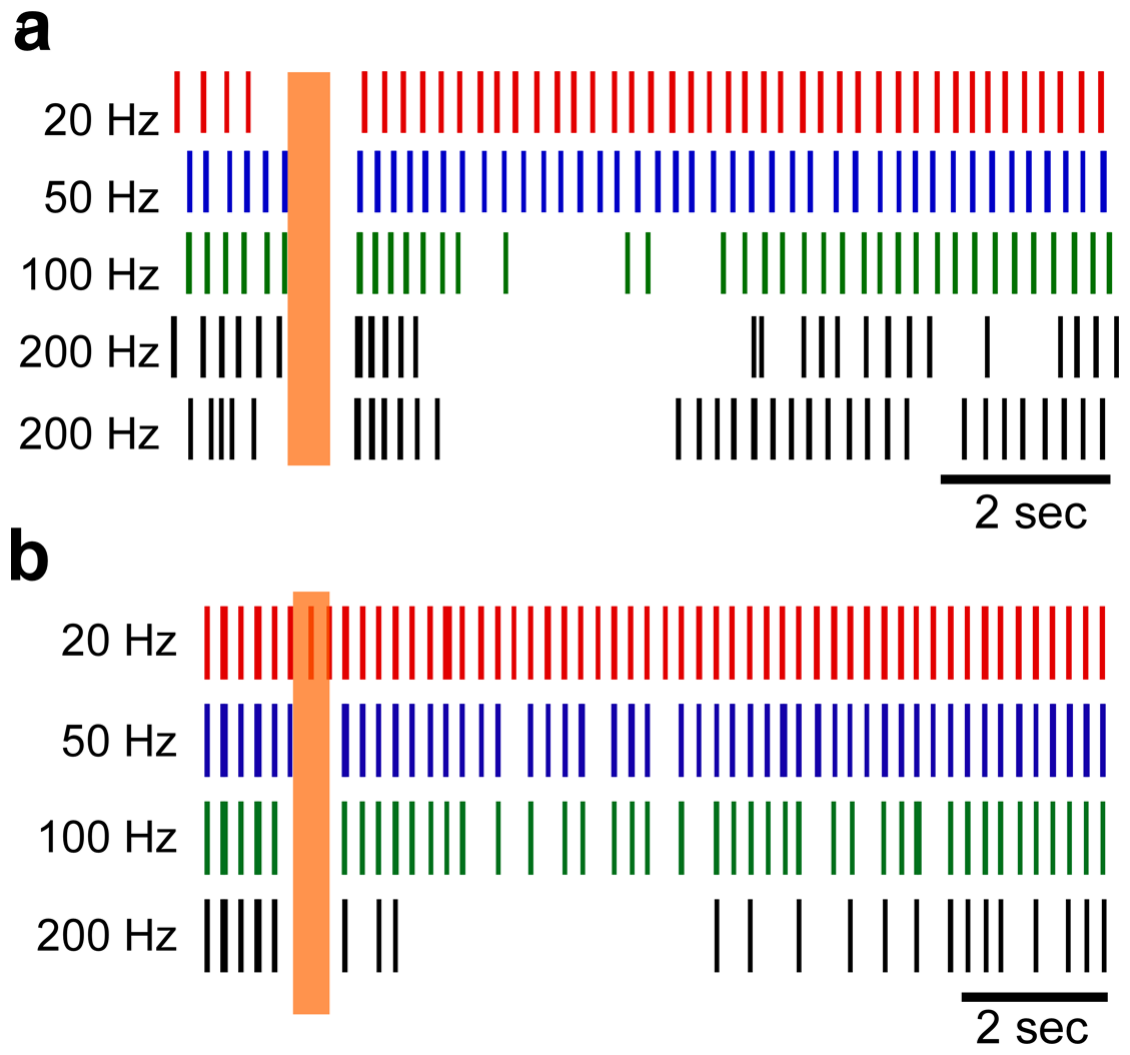
Individual neuron responses to changes in stimulus frequency. Rasters of an individual human thalamic neuron (**a**) and an individual model neuron (**b**) to various frequencies of stimulation for 0.5 seconds. The stimulus amplitude was 2 µA for the human response and 1.5 V for model response. Replicate trials of 200 Hz stimulation are shown to illustrate the trial-to-trial variability in the responses of the human neurons. The stimulus frequency for each raster is indicated at the left. The vertical shaded region indicates the time when stimulation was on.

We measured changes in neural responses to changes in the frequency of extracellular stimulation while the amplitude and duration of stimulation were held constant. The post-stimulus response of both human and model thalamic neurons exhibited a strong dependence on frequency, with the prolonged inhibitory period observed only at frequencies ≥100 Hz (**Fig. 5**) (linear least squares regression, human: *p*<0.02, R^2^ = 0.43; model: p<0.0001, R^2^ = 0.64). At frequencies less than 100 Hz, the pre-stimulus LTS bursting behavior resumed almost immediately after the cessation of stimulation.

**Figure 6:**
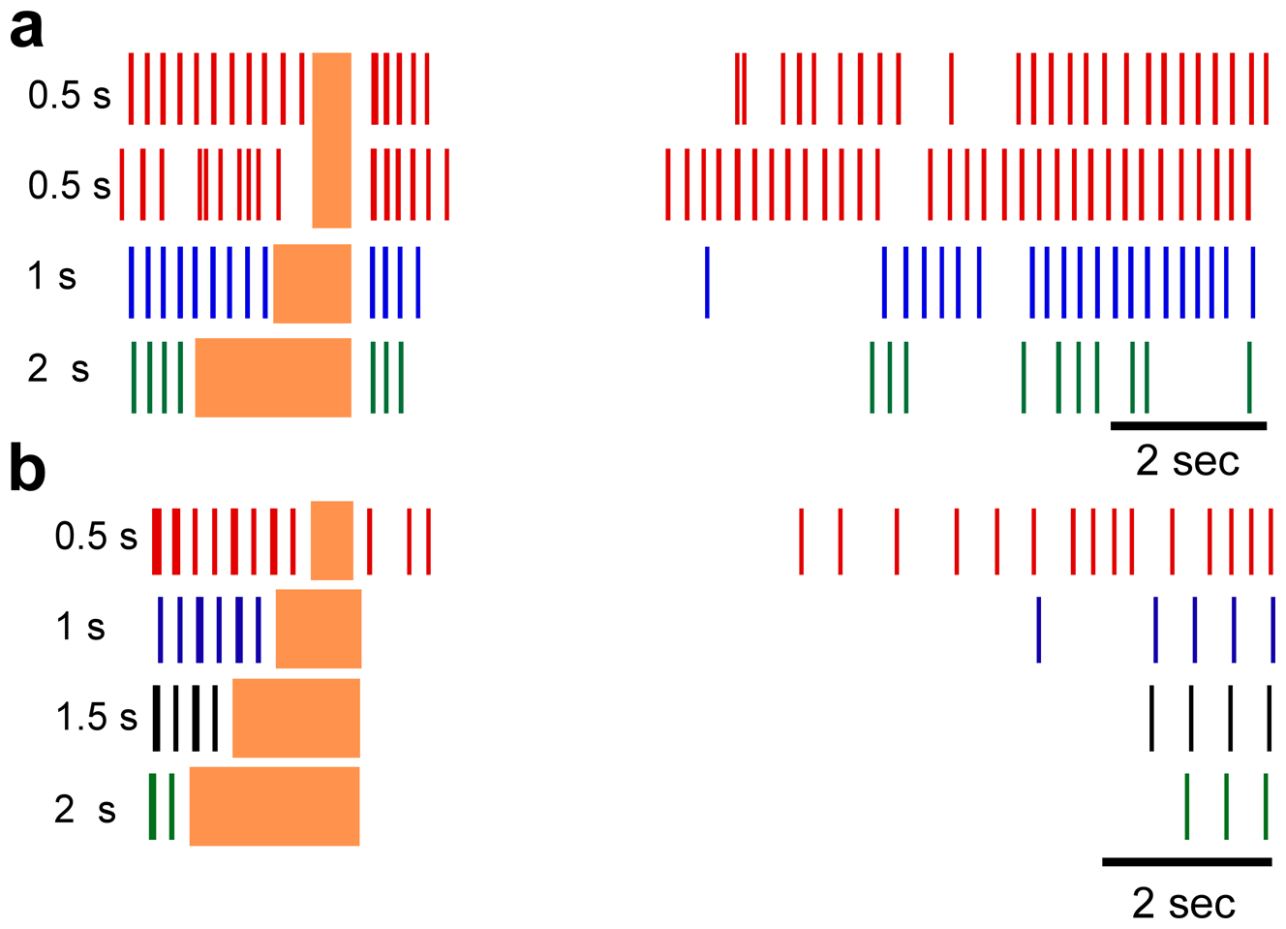
Individual neuron responses to changes in stimulus duration. Rasters of an individual human thalamic neuron (**a**) and an individual model neuron (**b**) to various durations of stimulation at 200 Hz. The stimulus amplitude was 2 µA for the human response and 1.5 V for model response. Replicate trials of 0.5 s stimulation are shown to illustrate the trial-to-trial variability in the responses of the human neurons. The duration frequency for each raster is indicated for each raster. The vertical shaded region indicates the time when stimulation was on, and the duration for each stimulation period is indicated to the left of each raster.

Finally, we measured changes in neural responses to changes in the duration of extracellular stimulation while the amplitude and frequency of stimulation were held constant. The post-stimulus response of human and model thalamic neurons demonstrated a moderate dependence on the duration of stimulation (**Fig. 6**). With longer stimulation durations, the human thalamic neurons exhibited a slight reduction in the number of post-stimulus burst epochs and an increased duration of the inhibitory period. There were not sufficient human thalamic recordings at stimulus durations other than 0.5 s to compute a linear least squares fit. The model thalamic neurons also exhibited a longer inhibitory period with longer durations of stimulation (*p*<0.0001, linear least squares regression, R^2^ = 0.19) (**Fig. 6b**). As with increases in stimulus amplitude, increasing the duration of stimulation led to a reduction in the initial post-stimulus burst response in model thalamic neurons.

### Impact of neuromodulator-dependent currents on post-stimulus model responses

**Figure 7:**
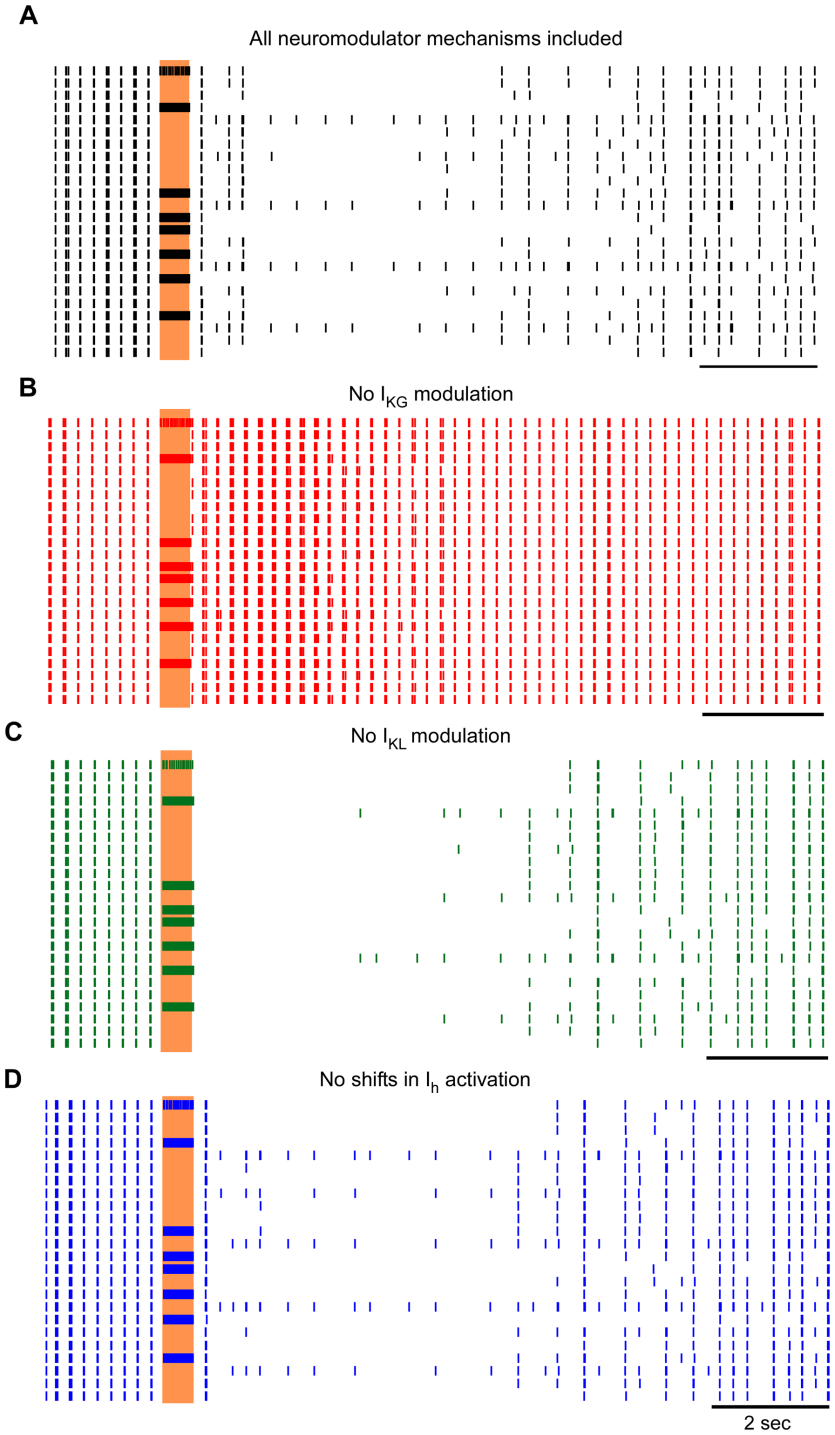
Impact of neuromodulator-dependent currents on post-stimulus model responses. Rasters of population of 24 model thalamic neurons in response to stimulation at 200**a**. Simulations were conducted with all of the neuromodulator-dependent mechanisms intact, and subsequently with each of the mechanisms removed individually from each simulation: **b**. neuromodulator-dependent activation of the pertussis toxin sensitive potassium current (I_KG_); **c**. Neuromodulator-dependent inhibition of a non-pertussis toxin sensitive leak potassium current (I_KL_); and **d**. Neuromodulator-dependent shifting of the activation curve of the hyperpolarization-activated cation current (I_h_). The vertical shaded region indicates the time when stimulation was on.

The effects of neuromodulator-dependent currents on the post-stimulus responses of model thalamic neurons were determined by independently removing each of the neuromodulator-dependent effects: 1) activation of the pertussis toxin sensitive potassium current (I_KG_), 2) inhibition of a non-pertussis toxin sensitive leak potassium current (I_KL_), and 3) shifting of the activation curve of the hyperpolarization-activated cation current (I_h_). Removing each neuromodulatory mechanism caused distinct effects on the post-stimulus responses of the model neurons (**Fig. 7**), but the effects were ubiquitous across the population of neurons. When neuromodulator impacts on I_KG_ were removed, the prolonged post-stimulus inhibition disappeared entirely (**Fig. 7b**). In contrast, when neuromodulator impacts on I_KL_ were eliminated, the prolonged post-stimulus inhibition remained intact, but the brief duration bursting prior to the inhibition no longer occurred (**Fig. 7c**). Finally, when neuromodulator impacts on shifts in activation of I_h_ were eliminated, the prolonged post-stimulus inhibition remained intact, but the degree of bursting prior to the inhibition was reduced (**Fig. 7d**).

**Figure 8:**
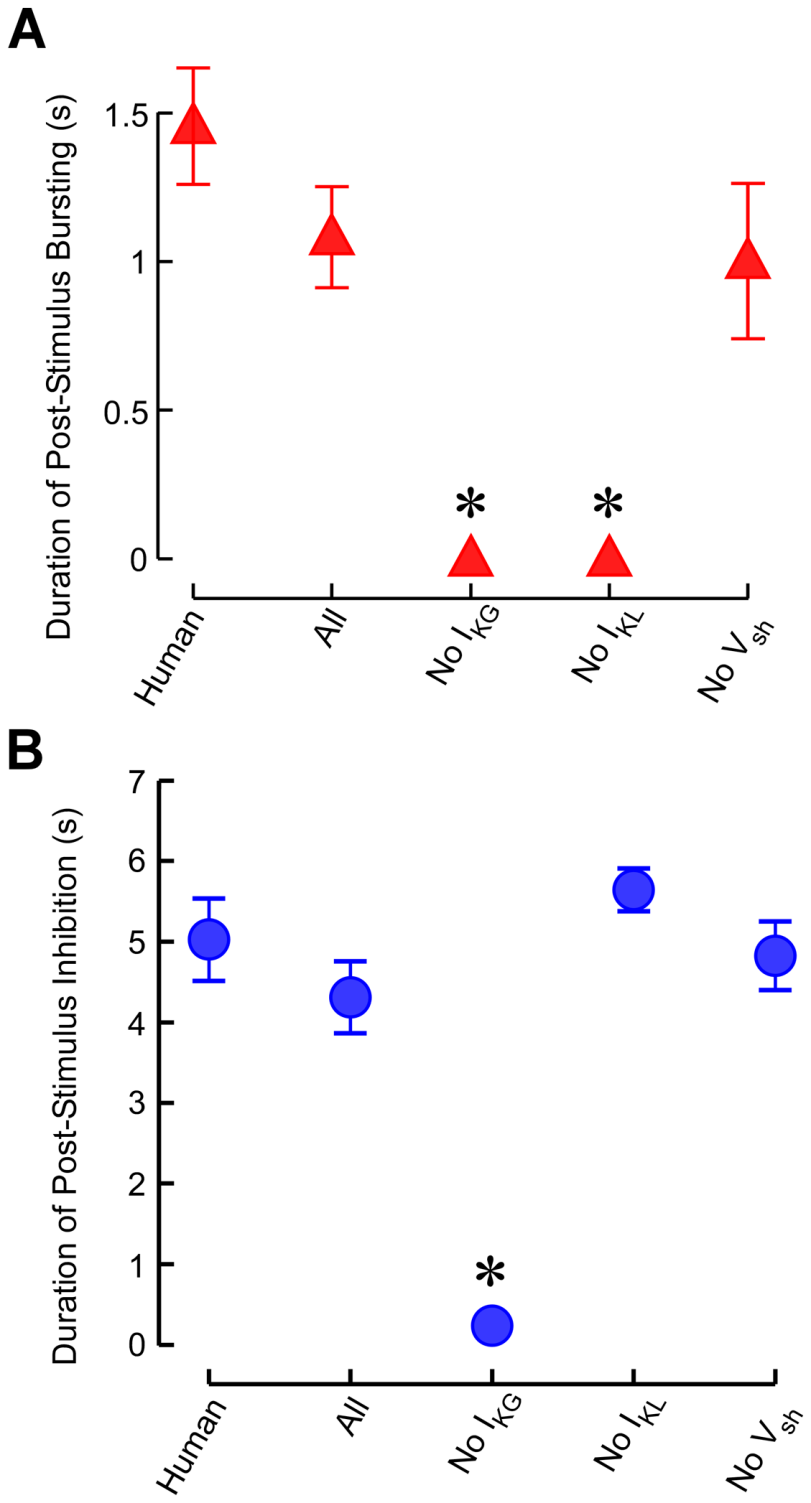
Changes in duration of the post stimulus bursting and inhibition. **a**. Duration of post stimulus bursting in human thalamic neurons and in model thalamic neurons under the four stimulus conditions. **b**. Duration of post stimulus inhibition in human and model thalamic neurons under the four stimulus conditions. Data are presented as mean ± s.e.m. *Mean is statistically different from the mean of the human thalamic neurons (*p*<0.05, Bonferonni multiple comparison).

We quantified changes in the duration of post stimulus bursting and prolonged inhibition for human and model neuronal responses (**Fig. 8**). The duration of the initial post-stimulus bursting was not significantly different between the responses of neurons in the human, the full model, and the model with no modulation of the shifts in I_h_ activation (**Fig. 8a, ***p*>0.05, Bonferroni multiple comparison after significant ANOVA: F_4,119_ = 16.2, p<0.0001). On the other hand, removing either I_KG_ or I_KL_ modulation caused dramatic reductions in the duration of post-stimulus bursting as compared to the other three conditions (*p*<0.05, Bonferroni multiple comparison). The duration of the post-stimulus inhibitory period was not significantly different between the responses of neurons in the human and the model for all conditions, except when I_KG_ modulation was removed (**Fig. 8b, ***p*>0.05, Bonferroni multiple comparison after significant ANOVA: F_4,119_ =  30.1, p<0.0001). Removing I_KG_ modulation caused a substantial reduction in the post-stimulus inhibitory period as compared to the other four conditions (*p*<0.05, Bonferonni multiple comparison).

### Impact of neuromodulator-dependent currents on model responses during stimulation

**Figure 9:**
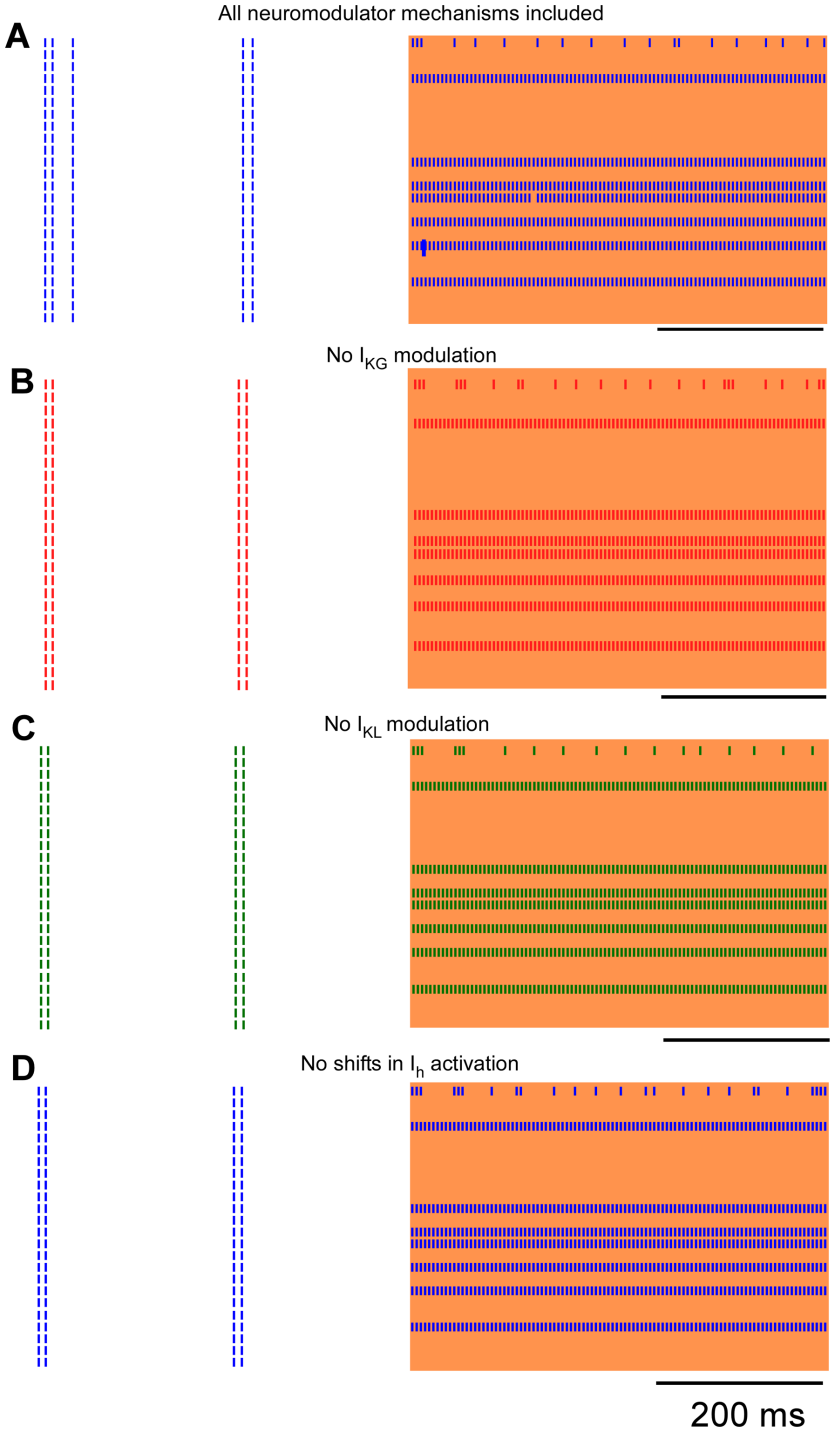
Impact of neuromodulator-dependent currents on responses of model thalamic neurons during stimulation. Rasters of multiple model neurons in response to stimulation at 200**Figure 7**, but the timeline is expanded to show the responses of model neurons during simulation. **a**. Simulations were conducted with all of the neuromodulator-dependent mechanisms intact, and subsequently with each of the mechanisms removed individually from each simulation: **b**. neuromodulator-dependent activation of the pertussis toxin sensitive potassium current (I_KG_); **c**. Neuromodulator-dependent inhibition of a non-pertussis toxin sensitive leak potassium current (I_KL_); and **d**. Neuromodulator-dependent shifting of the activation curve of the hyperpolarization-activated cation current (I_h_). The shaded region indicates the time when stimulation was on.

In out setup, stimulation and recording through the same microelectrode precluded recording of thalamic neuronal responses during stimulation in human, but responses of model thalamic neurons during stimulation were quantified. Despite the prominent effects of neuromodulator-dependent currents on the post-stimulus responses, the axonal output of model neurons during stimulation were largely independent of neuromodulator-mediated effects on ionic currents. Two response types were observed during stimulation across conditions: phase-locked firing and persistent inhibition (**Fig. 9**). Neurons that exhibited phase-locked firing during stimulation with the full complement of neuromodulator-dependent mechanisms intact continued to exhibit phase-locked firing when any of the neuromodulator-dependent mechanisms was disabled. Likewise, neurons that were inhibited during stimulation with the full complement of neuromodulator-dependent mechanisms intact were also inhibited during stimulation when any of the neuromodulator-dependent mechanisms was removed. Finally, one neuron responded with spikes every few stimulation pulses in a seemingly random fashion across all conditions.

## Discussion

We measured the responses of human and model thalamic neurons following cessation of short epochs of high frequency extracellular stimulation. Changes in the ionic conductance properties mediated by putative neuromodulators were essential to reproduce in model neurons the complex pattern of post-stimulus activity observed in human neurons. Specifically, modulation of the pertussis toxin sensitive potassium current I_KG_ was critical to the long pauses that occurred after stimulation, and modulation of the non-pertussis toxin sensitive leak potassium current I_KL_ was important for evoking the short epochs of bursts that preceded the long pauses. Further, the axons of the model TC neurons responded robustly to suprathreshold stimulation in the period during stimulation, and thus the influence of neuromodulators on the output of thalamic neurons during HFS may be largely masked by direct activation of their axons but nevertheless may contribute to blocking the transmission of pathological network oscillations.

The clinical effects of DBS in treating the motor symptoms of movement disorders are well established, but the mechanism(s) through which DBS exerts this influence are still unclear [18], [31]. The manner in which DBS alleviates symptoms bears close parallels with lesions in the same target nuclei, which led to early hypotheses that the therapeutic effects of DBS were due to inhibition of the target nucleus, although it was also proposed that DBS may reduce tremor by masking the low frequency neuronal oscillatory activity by generating high frequency continuous firing - termed “jamming” [32] or “informational lesion” [33]. The inhibitory hypothesis was supported by recordings of human thalamic, pallidal and subthalamic neurons in response to HFS, in which post-stimulus inhibition was demonstrated in the period immediately after the high frequency trains [15], [34], [35], as also seen in the present work and in recent results demonstrating frequency-dependent inhibition of neuronal firing during the stimulation train [36]. However, in apparent conflict with these results, animal studies indicated that the neuronal activity of at least some of the neurons in globus pallidus increased during high frequency stimulation of STN [37] and decreased in thalamus following pallidal stimulation (consistent with exciting the inhibitory GABAergic pallidal neurons) [38], and that the levels of glutamate and GABA were increased in SNr following STN stimulation [39]. Nevertheless, these findings are consistent with the dual effects of activation of the efferent axons while the somata may be inhibited [18] as revealed also in the findings from modeling in the present study and each effect may contribute to the final therapeutic effect. Additional studies indicated that efferent activity was not only activated, but also regularized (driven one-for-one by the stimulation pulse) by DBS when the frequency of stimulation was sufficiently high [33], [40], [41]. Furthermore, recent studies indicated that DBS may suppress tremor by masking burst driver inputs to the thalamus from the cerebellum, and that masking the pathological inputs, regardless of the resultant activity, was sufficient to suppress tremor [19].

The present study provides a logical explanation for these seemingly conflicting results. Specifically, both human neural recordings and computational modeling indicate that brief periods of HFS evoke periods of sustained inhibition following cessation of HFS. This is consistent with the hypothesis that high-frequency DBS inhibits the target nucleus. Additionally, model neurons showed a strong tendency toward excitation and regularization of the efferent axons *during* high-frequency stimulation consistent with the hypothesis of excitation and regularization. The potential resolution of these hypotheses lies in the dichotomous nature of the responses of neurons during stimulation (excitation) versus in the period immediately after stimulation (inhibition). Further, this dichotomy extends to differences between the response of neuronal somata (inhibition) and axons (excitation) during DBS [18].

One limitation of our study is that we were unable to determine which specific neuromodulators were responsible for the observed post-stimulus responses. Our human thalamic neuron recordings and model neuron simulations were not designed to isolate the identities or roles of individual neuromodulators, only to assess the net impact of neuromodulators on I_KG_, I_KL_, and I_h_. The net influence of these neuromodulators can activate a pertussis toxin sensitive potassium current (I_KG_) [7], [8], inhibit a non-pertussis toxin sensitive leak potassium current (I_KL_) [9], and/or shift the activation curve of the hyperpolarization-activated cation current (I_h_) [11], [12]. Indeed, the post-stimulation inhibition of spindle oscillatory activity observed in thalamic slices [6], [42], which is even longer than that described in the present report, could be reproduced in a computational model that included glutamate-mediated activation of I_h_. Additional studies are required to determine the contributions of any individual neuromodulator on the responses of thalamic neurons to HFS, for example by *in vivo* cyclic voltammetry [43]. Further, the release of neuromodulators by DBS and their influence on ionic conductances may mediate effects of DBS other than tremor suppression.

Another limitation of our study was that the methodology precluded recording of responses of human neurons during stimulation. While recordings from human thalamic neurons during HFS would certainly strengthen the findings derived from our computational model, the lack thereof does not diminish the value of the computational findings. There was very good concordance between the post-stimulus responses of the model and the responses recorded in humans, and our computational results are consistent with abundant experimental evidence in animals indicating that the efferent axons of the stimulated nucleus are activated by high frequency DBS. HFS in the GPi of healthy monkeys reduced the firing rate of 77% of recorded thalamic cells, an inhibitory target of GPi axons, suggesting activation of GPi efferent fibers [38]. Similarly, HFS in the STN of Parkinsonian monkeys increased the firing rates in the globus pallidus (GP) internal and external segments, excitatory targets of the STN, suggesting activation of STN efferent fibers [37]. Positron emission tomography (PET) studies have also shown increased activity in the ipsilateral GP during STN DBS in humans with PD [44], [45]. Unilateral 6-hydroxydopamine (6-OHDA) lesions in rats increased glutamate and GABA in the substantia nigra reticulata (SNr) and GP as compared to intact rats, and these levels did not decrease during STN HFS—as they would have if DBS caused inhibition of efferent activity [46].

It should also be noted that synapses might be unreliable and that the current model did not account for this behavior. However, during electrical stimulation, there are a large number of axon terminals stimulated simultaneously, and it is reasonable to assume that the overall impact of stimulation on the receptors can be modeled by reliable transmission. Indeed, *in vivo* recordings in GPi during STN DBS [37] and in thalamus during GPi DBS [38] suggest that post-synaptic activity is reliably generated in some neurons, even during long trains of stimulation. The synapses in the current model were not intended to represent individual cell-to-cell transmission, but rather they represent the aggregate input from one group of cells to another group of cells.

Another limitation of this study is that the model did not replicate every aspect of neuronal firing in the human models with full fidelity. For example, the number of spikes per burst and the intraburst frequency in the model contrasted those recorded in the human thalamic neurons, and many model neurons responded with single spike oscillatory spiking after the long-duration inhibitory period. Further, the initial post-stimulus burst response was observed consistently in human neurons but was muted in the model neurons as the amplitude, frequency or duration of stimulation was increased. However, the most salient features of the oscillatory responses of human thalamic neurons to brief duration stimulation were reproduced. Specifically, the short-term recovery, followed by longer duration inhibition, were demonstrated in the model. Additionally, while the model may not replicate fully the impacts of neuromodulators on oscillatory responses in thalamic neurons to brief epochs of stimulation, the model serves as a valuable tool to suggest that neuromodulators play a role in the post-stimulus responses of thalamic neurons.

## Conclusion

Our findings indicate that LTS bursting neurons in the thalamus responded to brief epochs of HFS with brief-duration bursting, followed by prolonged-duration quiescence. Computer simulation of model neurons indicated that changes in I_KG_ from stimulation-dependent release of neuromodulators play an important role in the prolonged inhibition following cessation of stimulation, while changes in I_KL_ play an important role in the short bursting epochs that immediately precede the quiescence. While the neuromodulator-dependent currents strongly influenced the post-stimulus behavior of thalamic neurons, the effect of HFS on axons during stimulation appeared independent of these currents. The axons of the model thalamocortical relay neurons responded robustly to suprathreshold stimulation in the period during stimulation, and activity in these axons was regularized during HFS. Taken together, our results suggest that the effects of neuromodulators on the output of thalamic neurons, while profound during the period after HFS, appear to be limited during HFS. Neurons that were inhibited during DBS with the full complement of neuromodulators intact were also inhibited when any of the neuromodulators was removed, and this inhibition was largely masked by direct activation of neuronal axons whether or not neuromodulators were included.

## Supporting Information

File S1 Detailed description and validation of the computational model of a thalamocortical neuron and its synaptic inputs, including the effects of neuromodulators.
(DOCX)
