# Supplementary material for: Response of Human Thalamic Neurons to High-Frequency Stimulation
Source: PLoS One. 2014 May 7;9(5):e96026. doi: 10.1371/journal.pone.0096026 (PMC4013084; doi:10.1371/journal.pone.0096026)
Supplement: File S1 — Detailed description and validation of the computational model of a thalamocortical neuron and its synaptic inputs, including the effects of neuromodulators. (DOCX) [file pone.0096026.s001.docx]

**SUPPORTING INFORMATION**

## Detailed model description

#### Thalamocortical relay neuron

We used computer-based models of thalamocortical relay neurons and their synaptic inputs to simulate the response of thalamic neurons to extracellular stimulation. The thalamocortical relay neuron included a cell body, 251 dendritic compartments, and a double-cable axon with 30 nodes of Ranvier. The thalamocortical cell geometry was derived from a 3-D reconstruction of a filled thalamocortical cell from rat ([Destexhe et al., 1998](#_ENREF_4)), and the axon diameter was selected as representative of those in ventrolateral thalamus ([Kultas-Ilinsky et al., 2003](#_ENREF_10)). The resting transmembrane potential of the TC neuron cell body was -69 mV, and the resting transmembrane potential of all axons was -70 mV.

#### Inputs to thalamocortical relay neuron

Each input axon had an intrinsic pattern of activity subjected to modification by extracellular stimulation. The cortical input (CTX) consisted of a 20 Hz Poisson train of suprathreshold pulses ([Deschenes and Hu, 1990](#_ENREF_2), [Person and Perkel, 2005](#_ENREF_22)). Inhibitory intrinsic activity of inputs from the internal segment of the globus pallidus (GPi) was based on the burst activity recorded in the GPi of human subjects with PD ([Magnin et al., 2000](#_ENREF_11)). Inhibitory input from reticular neurons (RN) resulted from synaptic excitation of RN from the cortical input and excitation of RN via feedback connections from the TC output ([Ando et al., 1995](#_ENREF_1), [Steriade et al., 1997](#_ENREF_25)).

Activation of input synapses on the TC neuron was evoked not only by the intrinsic activity of the input axons, but also by the effects of stimulation on the biophysically-modeled input axons. The input axons were modeled as having the same morphological and conductance properties as the axon of the TC neuron, with a slight adjustment made to the nodal slow potassium conductance from 0.07 to 0.08 S/cm^2^. Hence, the responses of the TC neuron depended on a combination of the intrinsic synaptic inputs, changes in the intrinsic synaptic activity evoked by stimulation of the input axons, and the direct effects of stimulation on the TC neuron.

The synaptic effects on TC neurons were modeled by applying either excitatory or inhibitory synaptic conductances to each of the dendritic and somatic compartments of each TC neuron, with the distribution of synapses based on electron microscopic reconstructions of glutamatergic and GABAergic terminals on cat ventral thalamic neurons (**Table S.1**) ([Sato et al., 1997](#_ENREF_23)). The CTX input axon activated both AMPA and NMDA glutamatergic synapses that were located on the intermediate to distal dendrites (**Table S.1**) ([Jones, 2007](#_ENREF_9), [Miyata, 2007](#_ENREF_19)). The effects of AMPA synapses were larger in amplitude, but briefer in duration than NMDA synapses ([Schwarz and Schmitz, 1997](#_ENREF_24), [Miyata, 2007](#_ENREF_19)). The GPi and RN input axons both drove inhibitory GABA_A_ and GABA_B_ synapses, with distributions concentrated closer to the cell body than the glutamatergic inputs (**Table S.1**) ([Sato et al., 1997](#_ENREF_23)). The strength of GABAergic conductances was adjusted to induce LTS-type bursting in the model neurons (**Table S.2**).

In addition to synapses formed by the terminating axons onto the thalamocortical relay neuron, we included 1:1 synapses at the following locations: thalamocortical axon to reticular nucleus axon ([Ando et al., 1995](#_ENREF_1)); cortical axon to thalamic interneuron axon ([Ando et al., 1995](#_ENREF_1)); and cortical input to reticular nucleus axon ([Ando et al., 1995](#_ENREF_1)). These 1:1 synapses were assumed to be reliable, with each spike in the terminating axon resulting in a postsynaptic current sufficient to produce an action potential at the proximal end of the postsynaptic axon. The terminating axons of these synapses were assumed to lie outside the volume affected by the extracellular potentials generated by stimulation, and we implemented virtual terminating axons at these synapses (**Fig. 1a**, light lines). These virtual terminating axons fired action potentials faithfully with the middle node of the corresponding biophysically-modeled presynaptic axons (**Fig. 1a**, bold lines), but after a time delay appropriate for conduction of the action potential down a separate branch of the axon. This allowed antidromic action potentials generated by stimulation to drive these synapses in conjunction with the intrinsic inputs to the biophysically-modeled axons ([Grill et al., 2008](#_ENREF_6)).

The kinetic schemes and parameters of the TC neuron and input axons were implemented following the bursting TC neuron as previously reported ([McIntyre et al., 2004](#_ENREF_18)), with to parameters detailed in **Table S.3**. As well, the strength of glutamatergic conductances was adjusted to approximate experimental postsynaptic potentials for AMPA- and NMDA-dominated synapses (**Table S.2**) ([Ando et al., 1995](#_ENREF_1), [Miyata, 2007](#_ENREF_19)).

We also modeled the effects of putative neuromodulators released by stimulated terminals, including adenosine, histamine, acetylcholine, serotonin, and noradrenaline ([McCormick, 1992b](#_ENREF_13), [Steriade et al., 1997](#_ENREF_25)). The neuromodulators activated a pertussis toxin sensitive potassium current (I_KG_) ([McCormick and Prince, 1987b](#_ENREF_16), [a](#_ENREF_15)), inhibited a non-pertussis toxin sensitive leak potassium current (I_KL_) ([McCormick, 1992](#_ENREF_12)b), and shifted the activation curve of the hyperpolarization-activated cation current (I_h_) ([McCormick and Williamson, 1991](#_ENREF_17), [Pape, 1992](#_ENREF_21)).

#### I_KG_: Potassium channel activated by GABA_B_ and putative neurotransmitters

First, we included a potassium current known as I_KG_, which is modulated by a pertussis toxin-sensitive G-protein that can be activated by either GABA_B_, muscarinic, or A1-adenosine receptors ([McCormick, 1992](#_ENREF_13)b). To implement this channel, we started with a GABA_B_ mechanism that allowed the release of GABA to summate when multiple presynaptic action potentials arrived within a short period of time to enhance the postsynaptic response to presynaptic bursting ([Otis et al., 1993](#_ENREF_20), [Destexhe et al., 1996](#_ENREF_3)). The kinetic scheme for the GABA_B_ mechanism was taken from Destexhe *et al.* ([Destexhe et al., 1996](#_ENREF_3)), and the effects of activating A1-adenosine and muscarinic receptors was modeled by adding a new equation to the kinetic scheme for this channel. The modified kinetic scheme was as follows:

 (S.1)

 (S.2)

, (S.3)

where ***R*** is the fraction of activated GABA_B_ receptor, ***S*** is the fraction of activated A1 and/or muscarinic receptor, **[*G*]** is the concentration of activated G-protein, **[*C*]** is the concentration of GABA in the synaptic cleft, and ***M*** represents a unitless time course of adenosine and/or acetylcholine in the synaptic cleft. When GABA_B_ receptors are activated, **[*C*]** changes instantaneously from zero to 1, and then returns to zero after 0.3 ms ([Destexhe et al., 1996](#_ENREF_3)). In a similar fashion, when A1 and/or muscarinic receptors are activated, ***M*** follows an alpha function (**Fig. S.1**):

 (S.4)

 (S.5)

**$M=b-a$**, (S.6)

where ***a*** and ***b*** are dummy variables used to construct the alpha function; ***w* =** 0.0035 ms^-1^ is a constant weight added to both ***a*** and ***b*** upon the arrival of each stimulus pulse; and **τ_1_** = 500 ms and **τ_2_** = 510 ms are the decay time constants associated with ***a*** and ***b***, respectively.

In **Equations S.1 – S.3**, the K_i_s are the kinetic rate constants for activating (odd) and deactivating (even) the receptors or G-proteins, and were set to the following values: K_1_=0.52 mM^-1^ms^-1^, K_2_=0.0013 ms^-1^, K_3_=0.098 ms^-1^, K_4_=0.033 ms^-1^, K_5_=0.1 ms^-1^, and K_6_=0.000167 ms^-1^.

In **Equation S.3**, *φ_ι_* is a unitless constant that represents the magnitude of the response of adenosine and/or acetylcholine terminals to extracellular stimulation in the volume near the *i-*th TC neuron. The value of *φ_i_* was set equal to twice the extracellular potential generated at the soma of cell *i* during stimulation at 1V.

#### Linear “leak” potassium current (I_KL_) inhibited by putative neurotransmitters

Activation of muscarinic acetylcholine, α_1_ adrenergic, and/or H_1_ histaminergic receptors results in the suppression of a relatively linear "leak" potassium current, I_KL_, leading to slow depolarization of the TC neuron ([McCormick, 1992a](#_ENREF_12),[b](#_ENREF_13)). I_KL_ suppression was modeled by altering the leak current in McIntyre, et al. ([McIntyre et al., 2004](#_ENREF_18)) as follows:

**$I_{KL}=\bar{g}_{KL}m(V_{m}-E_{k})$** (S.7)

, (S.8)

where **$\bar{g}_{KL}$** = 0.00016 S/cm^2^ is the maximal conductance of the leak current; and *m* is a state parameter that decreases with activation of muscarinic, α_1_, and/or H_1_ receptors following **Equation S.8**. In **Equation S.8**, *φ_ι_* and ***M*** have the same meaning and values as in **Equations S.2** – **S.6**; ***w_L_*** = 2 ms^-1^ is a constant weight added upon the arrival of each stimulus pulse; and τ_m_ = 600 ms is the decay time constant of ***m***. Due to the linearity of this current, a simple scaling of the maximal conductance by ***m*** was sufficient to produce lasting depolarization in the soma of the TC neuron.

#### Shifts in activation curve of I_h_ driven by putative neurotransmitters

Activation of β adrenergic, serotoninergic and H2 histaminergic receptors enhances the hyperpolarization-activated cation current I_h_ ([Steriade et al., 1997](#_ENREF_25)), while activation of A1 adenosine receptors inhibits I_h_ ([McCormick, 1992b](#_ENREF_13), [Pape, 1992](#_ENREF_21)). Enhancement and inhibition of I_h_ are driven by rightward and leftward shifts in the activation curve of this current, respectively ([McCormick and Williamson, 1991](#_ENREF_17), [Pape, 1992](#_ENREF_21)). I_h_ was modeled as:

**$I_{h}=g_{h}m^{3}(V_{m}+43)$** (S.9)

**$\tau_{m}=\frac{1}{\left[e^{\left\{-14.59-(0.086(V_{m}+V_{shift}))\right\}}+e^{\left\{-1.87+0.0701(V_{m}+V_{shift})\right\}}\right]}$** (S.10)

**$m_{\infty}=\frac{1}{1+e^{\left\{\frac{(V_{m}+V_{shift}+75)}{5.5}\right\}}}$** (S.11)

, (S.12)

where ***V_shift_*** determines the left/right shifts in the activation curve, g_h_ = 0.0015 S/cm^2^ is the maximal conductance, and ***V_sho_*** = 5 mV is the baseline shift used previously ([McIntyre et al., 2004](#_ENREF_18)). In **Equation S.12**, *φ_ι_* and ***M*** have the same meaning and values as in **Equations S.2** – **S.6**; ***w_h_*** = 1 mV/ms is a constant weight added upon the arrival of each stimulus pulse; and τ_m_ = 600 ms is the decay time constant of ***V_shift_***.

### Model validation

The computational model of ­­TC neurons and their inputs reproduced a variety of experimental results without changing biophysical parameters or ionic conductances across validation or experimental simulations. First, a single-cell version of the TC neuron, with no synaptic inputs, demonstrated responses to 60 ms depolarizing pulses that were similar to *in vitro* recordings of guinea pig thalamic slices ([Jahnsen and Llinas, 1984](#_ENREF_8)) under various levels of current injection (**Fig. S.2a**). The responses of the single-cell TC neuron to 45 ms hyperpolarizing pulses was also similar to those of the same thalamic slice neurons (**Fig. S.2b**) ([Jahnsen and Llinas, 1984](#_ENREF_8)). Finally, the model reproduced well the tonic bursts that occur in thalamic neurons during DC hyperpolarization (**Fig. S.2c**) ([McCormick and Pape, 1990](#_ENREF_14)).


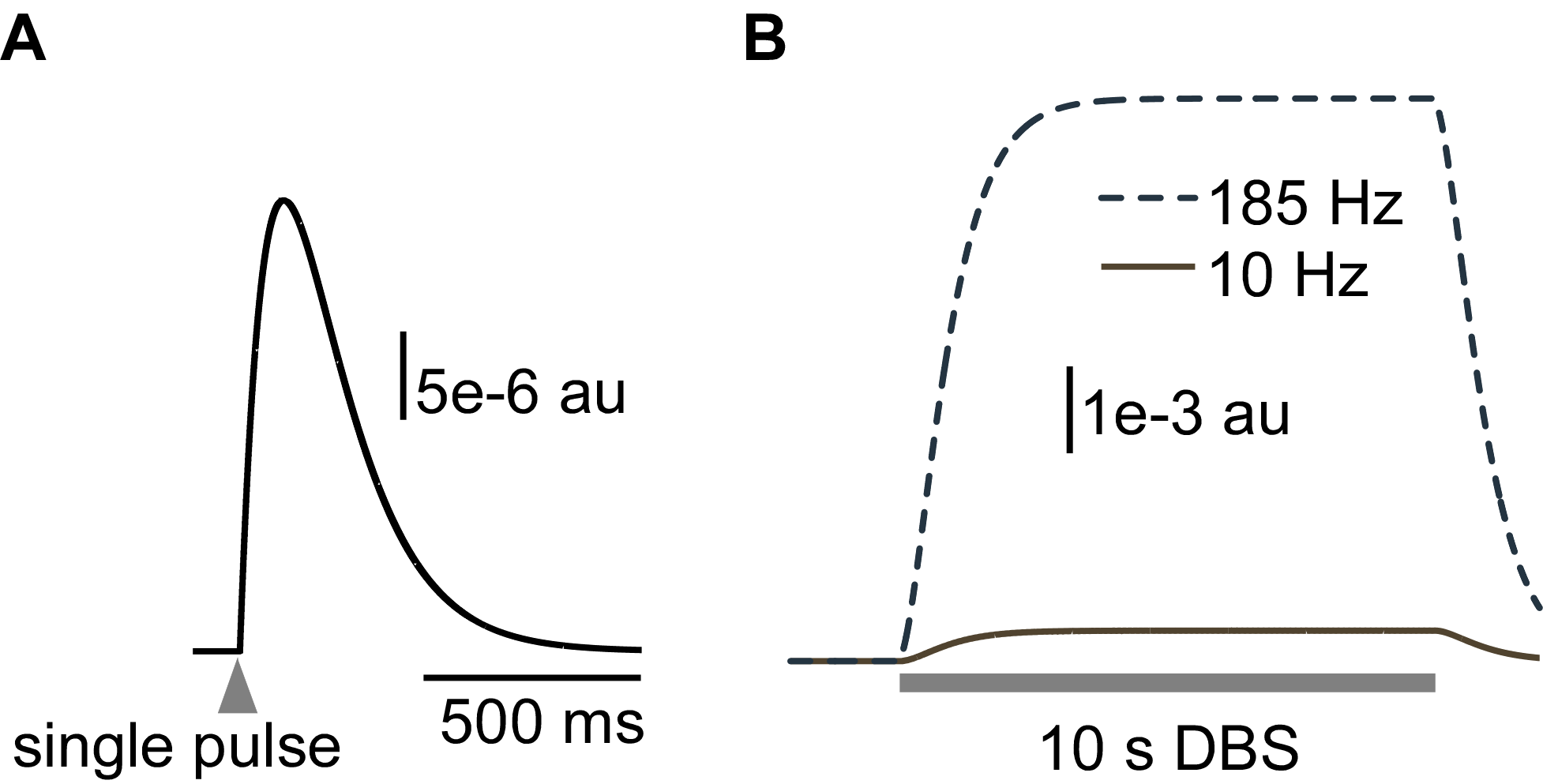


**Figure S.1. Response of *M* to stimulation.**

**a.** Time course of *M* in response to a single stimulus pulse (arrow) follows an alpha function. **b**. Time course of *M* in response to a 10 s epoch of stimulation at 10 Hz and 185 Hz. In these cases, the alpha function responses to individual stimulus pulses summate temporally and stabilize at different steady state amplitudes.


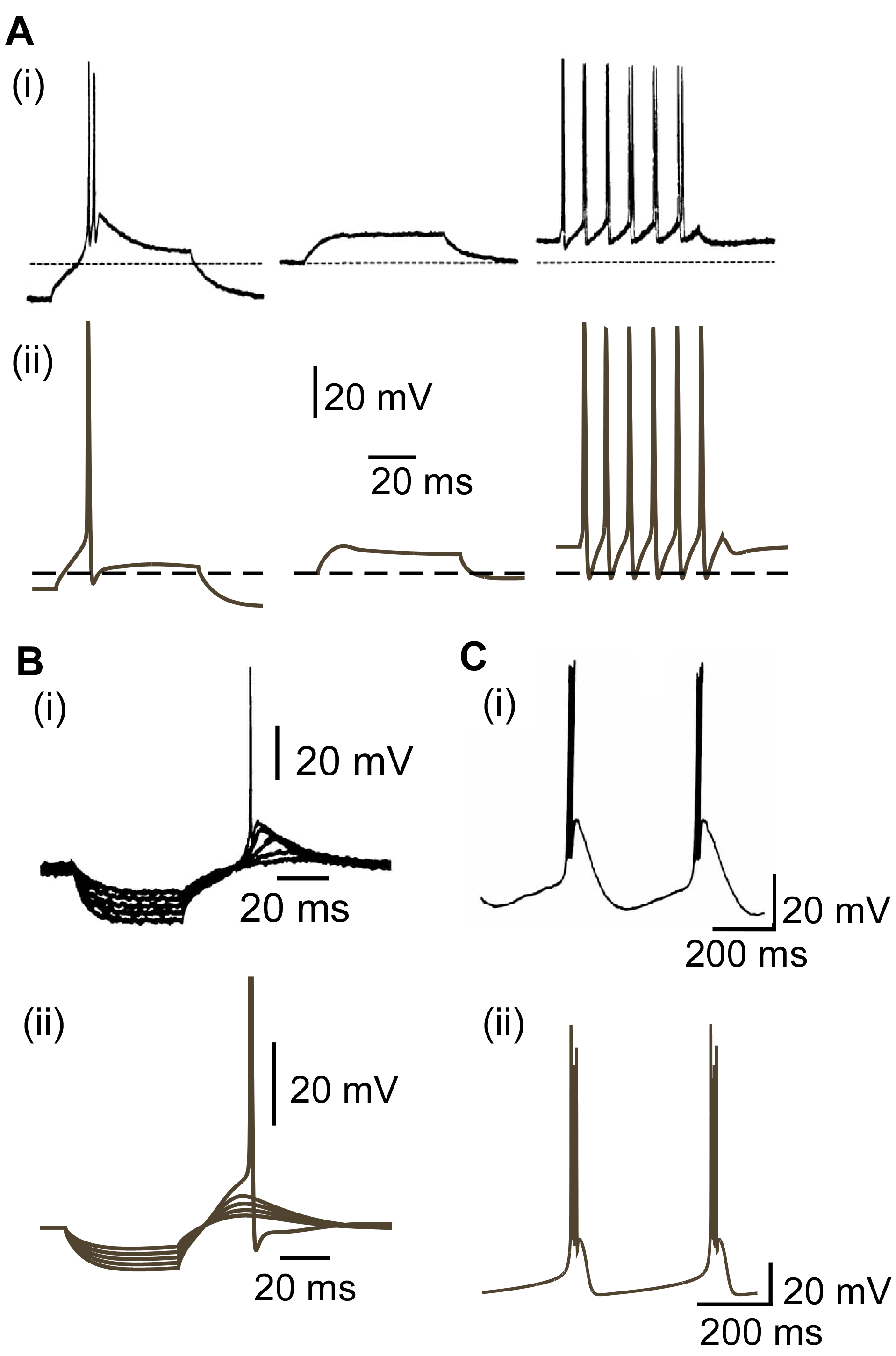


**Figure S.2. Comparison of responses of model and *in vitro* TC neurons.**

**a,** Responses to 60 ms depolarizing pulses were similar to those recorded in *in vitro* thalamic slices under various levels of DC polarization. (i) Reponses from thalamic neurons recorded from guinea pig slices in a hyperpolarized cell (left), a cell at rest potential (middle), and a depolarized cell (right) (Jahnsen and Llinas, 1984). (ii) Reponses of model thalamic neuron to a depolarizing pulse of 0.55 nA for 60 ms at hyperpolarized (left), rest (middle), and depolarized (right) potentials. **b,** Model rebound responses to 45 ms hyperpolarizing pulses was similar to those of the same thalamic slice neurons. (i) Responses of *in vitro* neurons (Jahnsen and Llinas, 1984) . (ii) Responses of the model TC neuron. **c,** Model responded to DC hyperpolarization with rhythmic bursting. (i) Responses of *in vitro* neurons (McCormick and Pape, 1990). (ii) Responses of the model TC neuron.

Table S.1. Distributions of cortical, cerebellar and GABA synapses on TC dendrites

| Compartments* | Synapse type | Percent of synapses in compartment of given type |
| --- | --- | --- |
| Primary dendritic & cell body | GABA | 100% |
| Primary dendritic & cell body | Cortical | 0% |
| Secondary dendritic | GABA | 62% |
| Secondary dendritic | Cortical | 38% |
| Distal dendritic | GABA | 26% |
| Distal dendritic | Cortical | 74% |

*Dendritic compartments were classified as follows. Sections between the soma and first bifurcation were defined as primary dendrites, while sections between the first and second bifurcations were defined as seconday dendrites, and any sections distal to the second bifurcations were defined as distal dendrites.

Table S.2. Synaptic conductance values

| Origin | Synapse type | Conductance (nS) |
| --- | --- | --- |
| Cortical | NMDA | 0.0164 |
| Cortical | AMPA | 0.00082 |
| RN | GABA_A_ | 0.0003 |
| TIN | GABA_A_ | 0.075 |
| RN | GABA_B_ | 0.002 |
| TIN | GABA_B_ | 0.005 |

Table S.3. Parameters altered from model published previously ([McIntyre et al., 2004](#_ENREF_18))

| Cell type affected | Compartments affected | Channel | Parameter | Previous value [or equation] | New value [or equation] |
| --- | --- | --- | --- | --- | --- |
| TC | soma/dendrites | T-type Ca^2+^ | P_CaT_ | 0.0001 cm/s | 0.000075 cm/s**^*^** |
| TC | soma/dendrites | T-type Ca^2+^ | τ_h_ for V_m­_ > -80 | **$9.33+0.333e^{-(V_{m}+25)/10.5}$** | **$1.6147\left[8.91+0.318e^{-(V_{m}+25)/10.5}\right]$^†^** |
| TC | soma/dendrites | T-type Ca^2+^ | τ_h_ for V_m­_ ≤ -80 | **$0.333e^{-(V_{m}+470)/66.6}$** | **$0.318e^{-(V_{m}+470)/66.6}$** |
| TC | soma/dendrites | T-type Ca^2+^ | τ_m_ | **$0.204+\frac{0.333}{e^{-(V_{m}+135)/16.7}+e^{-(V_{m}+19.8)/18.2}}$** | **$0.195+\frac{0.318}{e^{-(V_{m}+135)/16.7}+e^{(V_{m}+19.8)/18.2}}$** |
| TC | soma/dendrites | T-type Ca^2+^ | m_∞_ | **$m_{\infty}=1/\left[1+e^{\left\{-(Vm+60)/6.2\right\}}\right]$** | **$m_{\infty}=1/\left[1+e^{\left\{-(Vm+68)/6.2\right\}}\right]$^*^** |
| TC | soma/dendrites | Leak | g_NaL_ | 0.0000095 S/cm^2^ | 0.0000305 S/cm^2^**^**^** |
| TC | soma/dendrites/ initial segment | Slow K^+^ | I_Ks_ | **** | **^§^** |
| ALL | nodes | Slow K^+^ | β_s_ | 0.03/[1+exp((Vm+80)/-1)] | 0.03/[1+exp((Vm+45)/10)] **^‡^** |
| ALL | nodes | Slow K^+^ | g_Ks_ | 0.07 S/cm^2^ | 0.07 S/cm^2^ TC axon, 0.08 S/cm^2^ input axons |
| ALL | nodes | Leak | g_Lk_ | 0.005 S/cm^2^ | 0.007 S/cm^2^**^*^** |
| ALL | ALL | All K^+^ | e_K_ | -95 mV | ~-95 mV, [K^+^]_i_ = 106 and [K^+^]_o_ = 3 mM |

**^*^** Adjusted to facilitate tonic bursting during DC-hyperpolarization (**Fig. S.2c**) ([McCormick and Pape, 1990](#_ENREF_14)) and post-stimulus inhibition (**Fig. S.4d**) ([Dostrovsky and Lozano, 2002](#_ENREF_5)).

**^†^** Equation updated to ensure that τ_m_ curves for V_m_ ≤ -80 mV and V_m_ > -80 mV meet at V_m_ = -80 mV.

**^§^** Equation updated with correct proportions from earlier study ([Huguenard and McCormick, 1992](#_ENREF_7)).

**^‡^** Equation updated to correct τ_s_ and s_∞_ curves (α_s_ and β_s_ were previously both monotonic in same direction).

**^**^**This was equal to the conductance used in the tonically-active version of previous model.

## REFERENCES

Destexhe A, Neubig M, Ulrich D, Huguenard J (1998) Dendritic low-threshold calcium currents in thalamic relay cells. J Neurosci 18:3574-3588.

Kultas-Ilinsky K, Sivan-Loukianova E, Ilinsky IA (2003) Reevaluation of the primary motor cortex connections with the thalamus in primates. J Comp Neurol 457:133-158.

Person AL, Perkel DJ (2005) Unitary IPSPs drive precise thalamic spiking in a circuit required for learning. Neuron 46:129-140.

Deschenes M, Hu B (1990) electrophysiology and pharmacology of the corticothalamic input to lateral thalamic nuclei: an intracellular study in the cat. Eur J Neurosci 2:140-152.

Magnin M, Morel A, Jeanmonod D (2000) Single-unit analysis of the pallidum, thalamus and subthalamic nucleus in parkinsonian patients. Neuroscience 96:549-564.

Ando N, Izawa Y, Shinoda Y (1995) Relative contributions of thalamic reticular nucleus neurons and intrinsic interneurons to inhibition of thalamic neurons projecting to the motor cortex. J Neurophysiol 73:2470-2485.

Steriade M, Jones EG, McCormick DA (1997) Thalamus: Organization and Function (Vol. 1): Elsevier.

Sato F, Nakamura Y, Shinoda Y (1997) Serial electron microscopic reconstruction of axon terminals on physiologically identified thalamocortical neurons in the cat ventral lateral nucleus. J Comp Neurol 388:613-631.

Jones EG (2007) The Thalamus. New York: Cambridge University Press.

Miyata M (2007) Distinct properties of corticothalamic and primary sensory synapses to thalamic neurons. Neurosci Res 59:377-382.

Schwarz C, Schmitz Y (1997) Projection from the cerebellar lateral nucleus to precerebellar nuclei in the mossy fiber pathway is glutamatergic: a study combining anterograde tracing with immunogold labeling in the rat. J Comp Neurol 381:320-334.

Grill WM, Cantrell MB, Robertson MS (2008) Antidromic propagation of action potentials in branched axons: implications for the mechanisms of action of deep brain stimulation. J Comput Neurosci 24:81-93.

McIntyre CC, Grill WM, Sherman DL, Thakor NV (2004) Cellular effects of deep brain stimulation: model-based analysis of activation and inhibition. J Neurophysiol 91:1457-1469.

McCormick DA (1992a) Cellular mechanisms underlying cholinergic and noradrenergic modulation of neuronal firing mode in the cat and guinea pig dorsal lateral geniculate nucleus. J Neurosci 12:278-289.

McCormick DA (1992b) Neurotransmitter actions in the thalamus and cerebral cortex and their role in neuromodulation of thalamocortical activity. Prog Neurobiol 39:337-388.

McCormick DA, Williamson A (1991) Modulation of neuronal firing mode in cat and guinea pig LGNd by histamine: possible cellular mechanisms of histaminergic control of arousal. J Neurosci 11:3188-3199.

Pape HC (1992) Adenosine promotes burst activity in guinea-pig geniculocortical neurones through two different ionic mechanisms. J Physiol 447:729-753.

Otis TS, De Koninck Y, Mody I (1993) Characterization of synaptically elicited GABAB responses using patch-clamp recordings in rat hippocampal slices. J Physiol 463:391-407.

Destexhe A, Bal T, McCormick DA, Sejnowski TJ (1996) Ionic mechanisms underlying synchronized oscillations and propagating waves in a model of ferret thalamic slices. J Neurophysiol 76:2049-2070.

Jahnsen H, Llinas R (1984) Electrophysiological properties of guinea-pig thalamic neurones: an in vitro study. J Physiol 349:205-226.

McCormick DA, Pape HC (1990) Properties of a hyperpolarization-activated cation current and its role in rhythmic oscillation in thalamic relay neurones. J Physiol 431:291-318.

Huguenard JR, McCormick DA (1992) Simulation of the currents involved in rhythmic oscillations in thalamic relay neurons. J Neurophysiol 68:1373-1383.
